# Targeting few to help hundreds: JAK, MAPK and ROCK pathways as druggable targets in atypical chronic myeloid leukemia

**DOI:** 10.1186/s12943-018-0774-4

**Published:** 2018-02-19

**Authors:** Stefania Rocca, Giovanna Carrà, Pietro Poggio, Alessandro Morotti, Mara Brancaccio

**Affiliations:** 1 Department of Molecular Biotechnology and Health Sciences, University of Torino, 10126 Torino, Italy; 2 Department of Clinical and Biological Sciences, University of Torino, 10043 Orbassano, Italy

**Keywords:** Atypical myeloid leukemia, Ruxolitinib, Trametinib, Fasudil, MAPK, ROCK, JAK2, CSF3R

## Abstract

Atypical Chronic Myeloid Leukemia (aCML) is a myeloproliferative neoplasm characterized by neutrophilic leukocytosis and dysgranulopoiesis. From a genetic point of view, aCML shows a heterogeneous mutational landscape with mutations affecting signal transduction proteins but also broad genetic modifiers and chromatin remodelers, making difficult to understand the molecular mechanisms causing the onset of the disease. The JAK-STAT, MAPK and ROCK pathways are known to be responsible for myeloproliferation in physiological conditions and to be aberrantly activated in myeloproliferative diseases. Furthermore, experimental evidences suggest the efficacy of inhibitors targeting these pathways in repressing myeloproliferation, opening the way to deep clinical investigations. However, the activation status of these pathways is rarely analyzed when genetic mutations do not occur in a component of the signaling cascade. Given that mutations in functionally unrelated genes give rise to the same pathology, it is tempting to speculate that alteration in the few signaling pathways mentioned above might be a common feature of pathological myeloproliferation. If so, targeted therapy would be an option to be considered for aCML patients.

## Background

Atypical Chronic Myeloid Leukemia (aCML) is an aggressive and genetically heterogeneous disease for which no standard of care exists. The classification of aCML, which is included in the group of Philadelphia-negative myeloid neoplasms, has been a matter of debate for years. While the identification of the translocation t(9;22)(q34;q11) in a patient with accumulation of mature granulocytes and their precursors is sufficient for the diagnosis of Chronic Myeloid Leukemia (CML) [1, 2], the absence of this translocation is pathognomonic of Philadelphia-negative Myeloproliferative Neoplasms. Given the concomitant presence of myeloproliferation and myelodysplasia, the 2002 World Health Organization (WHO) classification of myeloid neoplasms places aCML under the category called myelodysplastic/myeloproliferative neoplasms (MDS/MPN) [3] and the 2008 and 2016 revisions of WHO criteria did not change the classification [4, 5]. The MDS/MPN group includes chronic myelomonocytic leukemia (CMML), aCML, juvenile myelomonocytic leukemia (JMML), MDS/MPN with ring sideroblasts and thrombocytosis and MDS/MPN unclassifiable (MDS/MPN-U). According to the 2008 WHO classification of myeloid neoplasms and acute leukemia, the absence of *BCR-ABL* and *PDGFRA*, *PDGFRB* or *FGFR1* rearrangements are minimal diagnostic criteria for aCML [4, 6]. However, the main feature characterizing aCML is the presence of neutrophilic leukocytosis and marked dysgranulopoiesis. Moreover, to fulfil the diagnostic criteria, the white blood count (WBC) should be ≥13 × 10^9^/L with ≥10% of immature granulocytes and ≤20% blasts in the blood and the bone marrow [4, 6]. These diagnostic guidelines have been then applied in different studies that analyzed histopathological features and clinical data available for similar types of myeloid neoplasia like Chronic Neutrophilic Leukemia (CNL) and MDS/MPN-U. These reports confirmed that WHO criteria were really suitable to distinguish aCML from similar diseases [7–11]. For what concern patients’ treatment, no standard of care exists. Hematopoietic stem cell (HSC) transplantation is always the best option when a matching donor is available. Without this possibility, patients can be considered for treatment with general drugs like hypomethylating agents, pegylated-interferon-α, hydroxyurea, and/or erythropoiesis stimulating agents or for enrollment in clinical trials with specific inhibitors (the case of ruxolitinib and trametinib will be discussed later in this review) [12]. However, patients’ survival, which has been analyzed in different studies with some differences, remains dismal. In an Italian cohort of 55 aCML cases respecting the WHO criteria, the median overall survival was 25 months [13], while in an US study of 65 patients it was found to be 12.4 months [11].

## Recurrent signaling pathways involved in myeloproliferation

A big effort has been made in the last decades to elucidate the molecular mechanisms leading to myeloproliferation. The identification of oncogenic mutations in signal transduction proteins pointed to the role of specific pathways in inducing excessive proliferation of myeloid lineages [14]. The subsequent development of mouse models carrying mutations found in patients and, conversely, the analysis of unexpected myeloproliferative phenotypes in genetically modified mice proved that the aberrant activation of these specific pathways plays a causal role in the onset of the pathology [15]. It came out that pathological myeloid proliferation is supported by few signaling pathways known to induce myelopoiesis by transducing signals from cytokines and growth factor receptors [16–19]. In this review we will primarily focus on three signal transduction pathways, the Janus kinase 2/signal transducers and activators of transcription (JAK2/STAT), the mitogen-activated protein kinase (MAPK) and the Rho associated coiled-coil containing protein kinase 1/2 (ROCK1/2) pathways. For all of them a role in myeloproliferation has been demonstrated by in vitro and in vivo studies and their involvement in human myeloproliferative diseases, including aCML, has been described [6, 14, 20, 21]. Moreover, inhibitors targeting signal transduction components of these pathways are already in clinical use and have the potential to be used for personalized treatment of aCML patients.

## The JAK2/STAT pathway

JAK2 is a tyrosine kinase that plays an essential role in myelopoiesis by transducing cytokine signals from several receptors, like receptors for erythropoietin (EPO-R), thrombopoietin (TPO-R) and granulocyte colony-stimulating factor (G-CSF-R). JAKs associate with cytoplasmic domains of different cytokine and growth factor receptors. The binding of extracellular ligands causes changes in the receptors that permit the associated intracellular JAKs to phosphorylate one another. Trans-phosphorylated JAKs then phosphorylate downstream substrates, including STATs. Activated STATs enter the nucleus and bind to specific enhancer sequences in target genes, thus regulating their transcription [22].

The mutation that causes the substitution V617F results in the activation of JAK2 signaling even without receptor stimulation, leading to ligand-independent granulocyte proliferation [20]. The *JAK2* V617F mutation is found rarely in aCML cases [23, 24], while it is frequent in Polycythaemia Vera (PV), Essential Thrombocythemia (ET) and Myelofibrosis (MF) [5]. Although infrequent, *JAK2* V617F mutated cases could benefit of the JAK2 inhibitor ruxolitinib, already in clinical use for the treatment of intermediate or high-risk MF [24, 25]. There are no standard treatment options for MF patients except for HSC transplantation or palliative cures. Of note, JAK2 is found activated in the majority of them, even in absence of the *JAK2* V617F mutation, which is present in 50% of the patients [26]. *JAK2* mutational status or allele burden have been related to clinical signs of the disease like splenomegaly, transformation to Acute Myeloid Leukemia (AML) and overall survival [27–29], thus pointing to JAK2 inhibition as a promising strategy to treat MF. After a first study which evaluated the efficacy of ruxolitinib in preclinical models of *JAK2* V617F positive MPN [28], a phase I-II [30] and two phase III clinical trials (COMFORT I and II) were carried out with positive results [31, 32]. In the first case, 153 patients with *JAK2* V617F positive or *JAK2* V617F negative primary MF, post–essential thrombocythemia MF, or post–PV MF were enrolled. 44% of them showed reduction of splenomegaly and the majority of them, who received the drug at a dose of 10 mg twice daily to 25 mg twice daily, had more than 50% improvement in total or individual symptom scores according to the Myelofibrosis Symptom Assessment Form (MFSAF) [30]. In both COMFORT I (ruxolitinib vs. placebo) [29] and COMFORTII (ruxolitinib vs best available therapy) studies [33], patients receiving oral ruxolitinib showed reduced splenomegaly at week 48 and an improvement of debilitating symptoms and quality of life [30, 32, 34–37]. 5-years follow up analysis showed an advantage in terms of overall survival for both COMFORTI and COMFORTII studies: medium overall survival was not reached for ruxolitinib, while it was 3.8 years for placebo group [36] and 4.1 years for the group receiving the best available treatments [37]. Moreover, ruxolitinib has also been used in phase III clinical trials with patients affected by PV intolerant or resistant to hydroxyurea demonstrating an effectiveness in reducing splenomegaly and clinical symptoms [38–41]. However, ruxolitinib treatment induces a complete response only in a small percentage of patients [40, 41]. Concerning ET patients intolerant or resistant to hydroxycarbamide, ruxolitinib did not improve treatment efficacy in comparison with the best available therapy [42–44]. This result suggests that the effectiveness of targeted treatments depends not only on the presence of specific mutations, but also on the peculiar features of the pathology.

The lack of a complete response in MPN patients treated with ruxolitinib might be due to the activation of collateral oncogenic pathways, like the one of c-Jun N-terminal kinase (JNK) or PI 3-kinase (PI3K)/AKT serine/threonine kinase (AKT)/ mammalian target of rapamycin (mTOR) pathway [45]. Consistent with this, PI3K, AKT and mTOR inhibitors have been tested alone or in combination with ruxolitinib demonstrating synergistic effects in MPN cells [46–50]. STAT5 plays a crucial role in JAK2-driven myeloproliferation by inducing the expression of proteins promoting cell division, cytokines independent growth and cell survival like c-MYC, CYCLIN D2, ID1, BCL-XL and MCL-1 [51]. In vivo experiments demonstrated that JAK2 V617F requires STAT5 to induce MPN in mice, while STAT3 was found to be dispensable [52]. However, STAT5 specific inhibitors are not yet suitable for clinical applications [53]. It has been shown that JAK2 and PI3K/AKT/mTOR regulate STAT5 activation by inducing its phosphorylation on different residues and that ruxolitinib is ineffective in reducing STAT5 phosphorylation driven by the PI3K pathway [54]. Indeed, combined inhibition of JAK2, PI3K and mTOR in *JAK2* V617F mutated cells causes reduction of both JAK2 and PI3K mediated STAT5 phosphorylation, impairment of the clonogenic potential of *JAK2* V617F-mutated hematopoietic progenitors cells and reduced splenomegaly and myeloid cells infiltration in *Jak2* V617F knock-in mice [54]. These studies suggest the importance of PI3K/AKT/mTOR axis in myeloproliferative diseases; however, the effects of the inhibition of these molecules in aCML pre-clinical models and patients still need to be evaluated.

**Fig. 1:**
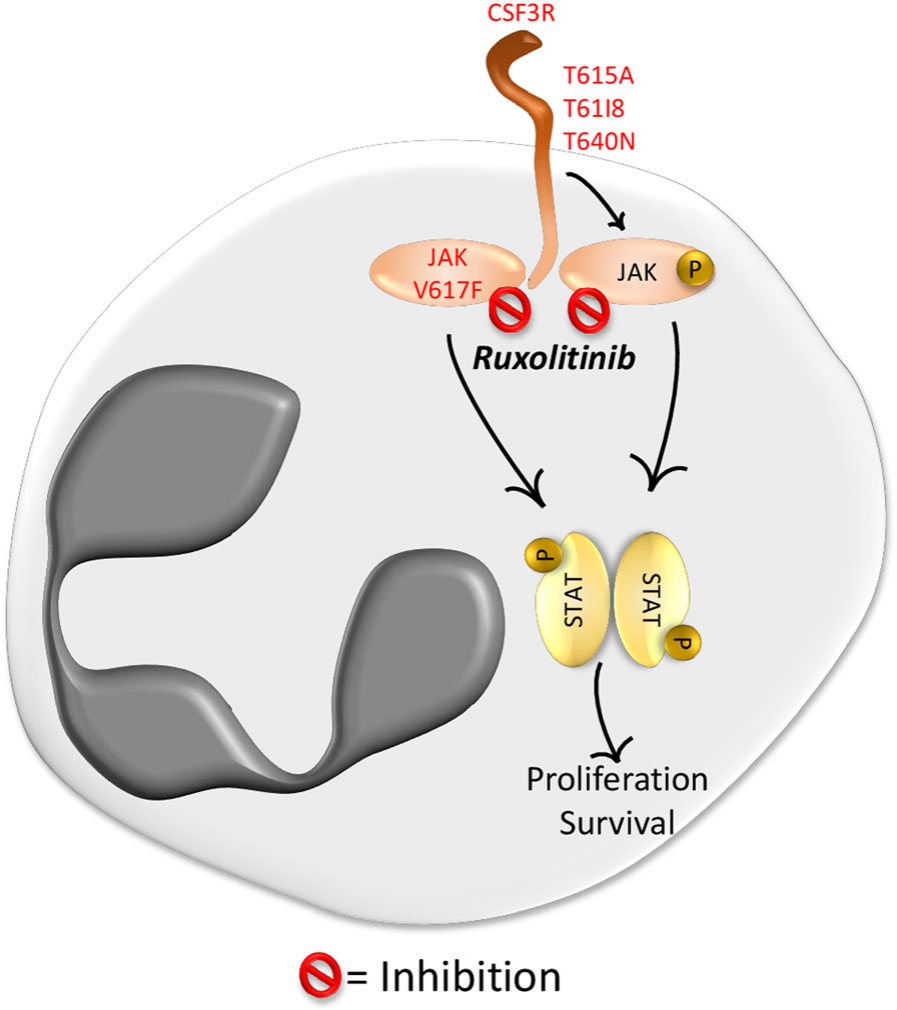
*JAK2* and *CSF3R* mutated patients can benefit of ruxolitinb treatment. In basal conditions, JAK2 signaling is initiated by the binding of cytokines to the associated receptors. Once activated, JAK phosphorylates STAT proteins inducing their dimerization and translocation to the nucleus, where they activate or suppress gene transcription. In the presence of *JAK2* V617F mutation, the JAK/STAT pathway is constitutively activated. CSF3R is known to signal through the JAK tyrosine kinase pathway. CSF3R membrane proximal mutations, such as T615A, T618I and T640 N, constitutively activate JAK-mediated signaling and are sensitive to its kinase inhibitor ruxolitinib

Even in absence of *JAK2* mutations, MPN cells use different strategies to induce JAK2 hyperactivation and trigger myeloid expansion [15]. It has been shown that *CALR* gene, encoding for calreticulin, is mutated in the vast majority of *JAK2* V617F negative MPN patients [55, 56]. Calreticulin is a Ca^++^ binding protein with chaperone activity located in the endoplasmic reticulum [56, 57]. Mutant calreticulin acquires the ability to bind to the thrombopoietin receptor (MPL receptor) in the ER and then on the cell surface, inducing ligand-independent activation of the JAK2/STAT/PI3K and MAPK pathways [15]. However, *CALR* mutations have been found rarely in aCML patients [11, 55, 56]. Mutations in genes coding for other JAK2 activators, like the TPO-R [58] and G-CSF-R, have been found in myeloproliferative disorders. *CSF3R* gene encodes for the Granulocyte colony-stimulating factor receptor (G-CSF-R), the more relevant JAK2-upstream regulator in aCML. This receptor provides the signal for growth and differentiation of granulocytes through the binding to its ligand: the granulocyte colony-stimulating factor 3 (G-CSF; CSF3) [59–61]. Two types of mutations were originally identified in a cohort of 27 patients with CNL or aCML [62]: membrane proximal mutations (T615A and T618I), which confer ligand-independent growth, and nonsense or frameshift mutations, resulting in the truncation of the cytoplasmic region with consequent alteration of granulocytic differentiation and proliferation [62–64]. Besides the JAK/STAT pathway [65, 66], G-CSF-R also signals through the tyrosine-protein kinase SYK and the SRC family kinase (SFK) LYN [67, 68]. When receptors carry truncating mutations, they signal through SFKs rendering the cells sensible to the multikinase inhibitor dasatinib. In contrast, when carrying the membrane proximal mutations, the receptor signals through the JAK/STAT pathway and in this case cells are sensitive to ruxolitinib [62]. In a first report, Maxson and colleagues found *CSF3R* mutations in 59% of patients with CNL or aCML, while subsequent analysis indicated that the activating *CSF3R* T618I mutation is present in < 10% of cases of aCML [11, 69, 70]. Thanks to these studies, the identification of *CSF3R* T618I in the context of neutrophilic leukocytosis is now strongly associated with a diagnosis of CNL, where it is present in approximately 80% of patients [69]. In line with this evidence, mice transplanted with hematopoietic cells expressing *CSF3R* T618I develop a CNL-like disease characterized by neutrophil expansion in the peripheral blood and bone marrow and neutrophil infiltration in spleen and liver [71]. Administration of ruxolitinib to these mice results in reduction of WBC, decreased spleen weight and increased body weight [71]. Another report described the acquisition of *CSF3R* mutations (both the proximal mutation T618I and a truncating mutation Q739*) in a patient progressing from MPN unclassifiable to aCML [72]. An alternative membrane proximal mutation, the T640 N, was described in a patient with MDS progressing to aCML-like disorder. This mutation confers ligand-independent growth, mimicking the *CSF3R* T618I, and sensitivity to ruxolitinib treatment [73]. The potentiality of ruxolitinib for *CSF3R* T618I-mutated patients was demonstrated with two case reports: a 75-years old man and a 11-years old girl with aCML. The man, who was refractory to hydroxyurea treatment, displayed decreased WBC, reduction of the spleen volume, increased haemoglobin and platelets count after ruxolitinib treatment [74]. The young girl received ruxolitinib for 8 weeks and the good response to the treatment allowed her to be bridged to allogenic HSC transplantation [75]. Currently, a phase II study (NCT02092324) is evaluating the efficacy of ruxolitinib treatment in patients with CNL or aCML. These evidences suggest that the evaluation of the presence of *JAK2* or *CSF3R* mutations in aCML patients could open the way for specific therapeutic interventions (Fig. 1).

## The RAS pathway

RAS proteins are small GTPases involved in signal transduction and frequently mutated in human cancers. There are three major isoforms (*HRAS*, *KRAS*, *NRAS*) which, in their GTP-bound form, signal through the MAPK and the PI3K pathways [76], promoting cancer cell survival and proliferation. Mutant RAS recruits RAF to the plasma membrane and activates the downstream Mitogen-activated protein kinase kinase (MEK)/ Mitogen-activated protein kinase 3 (ERK) signaling cascade [77]. Mutations in the *KRAS* and *NRAS* genes (but rarely in the *HRAS* gene) are frequently identified in myeloid disorders (15%–60%), including AML [78, 79], aCML [11], CMML [80] and JMML [81]. For decades scientists tried to develop effective strategies to target *RAS*-mutant cancers even if targeting RAS itself has proven to be difficult [77]. One of the attempts was to use nucleotide analogs to trap mutant RAS in an inactive state but this approach failed because of the high concentrations of GTP that make competition impossible. Screenings for compounds that restore GTPase hydrolysis to mutant RAS, in the presence or absence of GAP, also failed [77]. Given the difficulties in targeting RAS, many researches focused on inhibiting downstream effectors in the MAPK pathway. B-RAF has been tested as molecular target especially in solid tumors, as melanoma, where it is mutated in 50% of the cases. Therapies with RAF inhibitors lead to some degree of tumor regression, but poor response or acquired resistance are also common [82, 83]. Resistance is often due to a paradoxical activation of ERK, mainly in *RAS*-mutant cancers [84–86]. The results obtained with RAF inhibitors, led to the postulation that MEK1/2 are better therapeutic targets in RAS mutated hematological malignancies. Oncogenic RAS is sufficient to initiate myeloid leukemogenesis in mice: the expression of mutant K-RasG12D protein from the endogenous murine locus rapidly induces a fatal myeloproliferative disorder with 100% penetrance [87]. The efficacy of MEK inhibitors on myeloid *NRAS/KRAS* mutated leukemic cells have been tested using two different mouse models: a *Mx1-Cre, KrasLSL-G12D* mice, which develop a fatal myeloproliferative neoplasm [88] and mice transplanted with *NRAS* mutated AML cells (NrasG12D AML cells) [89]. In the first study mice were treated with the MEK inhibitor PD0325901 [90] which prolonged survival and reduced leukocyte count, anemia and splenomegaly [88]. In the second study, mice were treated with two different MEK inhibitors: PD0325901 and trametinib (also known as GlaxoSmithKline 1,120,212). Trametinib is an oral, selective and allosteric inhibitor of MEK1/MEK2 approved by the US Food and Drug Administration as a single agent or in combination with the B-RAF inhibitor dabrafenib (Tafinlar; GlaxoSmithKline) for the treatment of unresectable or metastatic melanoma with a *BRAF* V600E/V600 K mutation [91]. Both MEK inhibitors significantly improved the survival of recipient mice by inhibiting AML proliferation [89]. Trametinib efficacy was then tested in an open-label, dose-escalation, nonrandomized, multicentre phase 1/2 study (GlaxoSmithKline study MEK111759; ClinicalTrials.gov identifier NCT00920140) [92]. 97 patients (AML, 75%; high-risk MDS, 12%; CMML, 11%; and ALL, 1%) were enrolled in the study: 13 patients had *KRAS* mutations, and 54 patients had *NRAS* mutations. Among *RAS* mutated patients, the overall response rate was 21% with reduction in bone marrow and peripheral blasts. However, the response did not translate into survival advantage, probably due to the fact that *RAS* mutations emerge late during leukemogenesis and that many of the patients enrolled were already resistant to previous therapies, possibly due to the presence of different subclones with various levels of dependence on the MAPK pathway [92]. For what concern aCML, *KRAS/NRAS* mutations were identified in 7/20 patients (35%) [11]. A case report described a 81-years-old male with heterozygous NRAS G12D mutation who, after receiving trametinib, showed improvements in his blood count and a durable disease control for 14 months of follow-up [93].

**Fig. 2:**
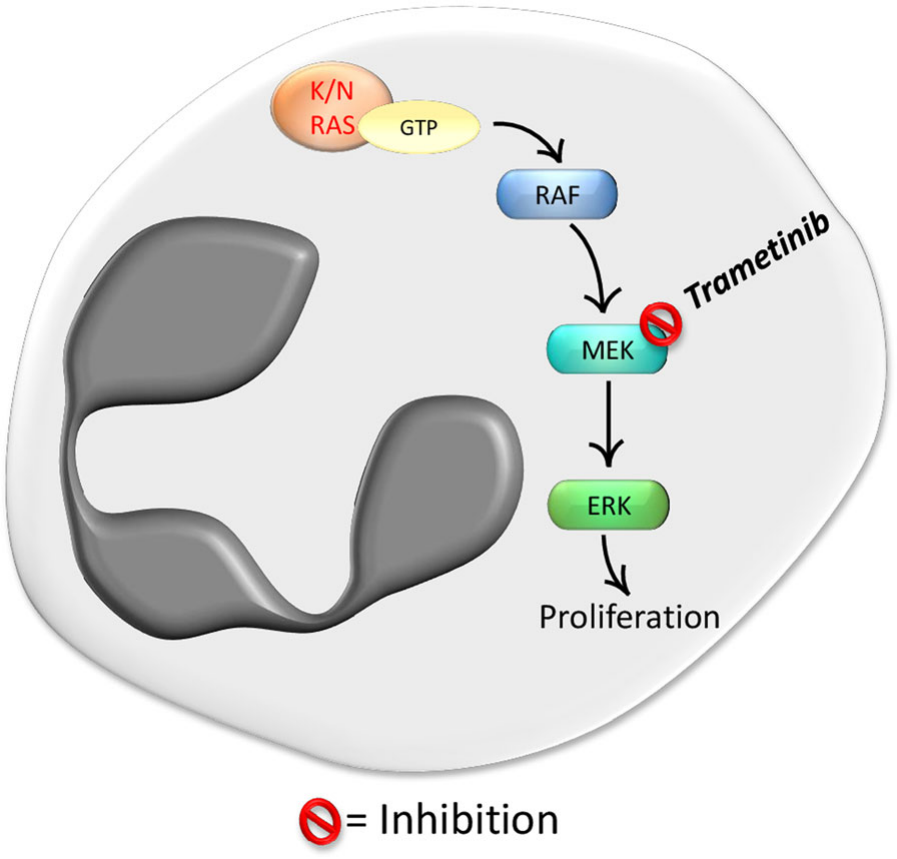
Targeting RAF–MEK–ERK signaling pathway. GTP-bounding RAS recruits and activates RAF, which in turn initiates a cascade of protein phosphorylation starting with MEK. Activated MEK phosphorylates ERK that moves from the cytoplasm to the nucleus where it phosphorylates several transcription factors. Mutational activation of RAF–MEK–ERK cascade contributes to progression of the disease. Selective inhibitors of MEK, e.g. trametinib, cause potent and durable suppression of ERK signaling

This unique clinical report on an aCML patient treated with trametinib, together with the results obtained in preclinical studies, highlight the need for clinical trials to test the efficacy of MEK inhibitors in larger cohorts of *RAS*-mutated aCML patients (Fig. 2). Of note, RAS also activates PI3K/AKT/mTOR, which can represent an axis promoting proliferation and survival of cancer cell. For this reason, combined treatment using both MEK inhibitors and PI3K/AKT/mTOR inhibitors could be more effective in inducing tumor regression and many clinical trials are already testing this possibility in different contexts [94]. Interestingly, treatment with GDC-0941, an orally available inhibitor of class I PI3K isoforms, extended *Mx1-Cre, KrasLSL-G12D* mouse survival, inducing reduction of anemia, splenomegaly and leucocytosis, by inhibiting simultaneously MAPK and PI3K signaling [95].

## The ROCK pathway

**Fig. 3:**
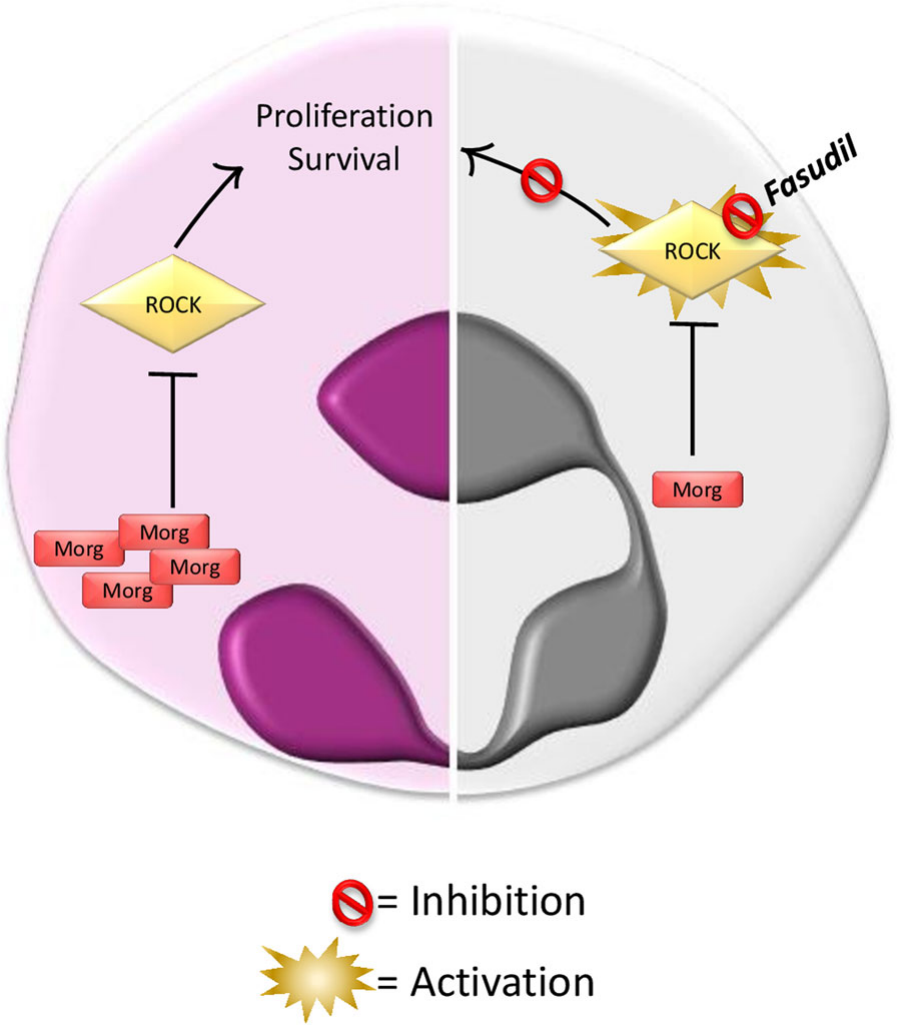
Fasudil treatment for Morgana^low^ leukemic cells. A diminished Morgana expression induces ROCK hyperactivation. ROCK plays a key role in multiple cell signaling processes, inducing proliferation and survival in myeloid cells. ROCK inhibition, through fasudil, results in increased apoptosis of leukemic cells

ROCK1/2 are two multifunctional proteins with 65% of overall identity [96]. They play different roles in the cells: from regulating cytoskeletal rearrangements to taking part in signaling pathways leading to apoptosis and proliferation [97–105]. ROCK signaling deregulation is emerging as a key feature in myeloid leukemias. Mali and colleagues demonstrated that ROCK is constitutively activated in cells harboring oncogenic forms of KIT, FLT3, and BCR-ABL [101]. ROCK inhibition by Y-27632 or H-1152 strongly reduces leukemic cell proliferation [21, 101, 106, 107] demonstrating that mutant tyrosine kinase receptors are able to induce leukemic transformation, at least in part, through ROCK signaling. A subsequent report demonstrated that ROCK downregulation strongly impairs cell proliferation also in human CD34+ AML cells. In fact, if primary AML cells silenced for ROCK are xenotransplanted in mice, human chimerism is strongly reduced compared to control cells, demonstrating that the ROCK activity is required for oncogenic proliferation in vivo [108]. Data from our laboratory added a little piece to the puzzle by discovering the role of morgana, an ubiquitous protein coded by the *CHORDC1* gene, in myeloproliferation [100, 109–111]. While *Chordc1* knock-out mice die early during embryogenesis, heterozygous mice are vital, but develop spontaneously a fatal and transplantable myeloproliferative disease resembling human aCML [15, 112]. Morgana is a chaperone protein able to inhibit both ROCK1 [100] and ROCK2 [110], and recently found to be required for NF-κB signaling [113]. *Chordc1* heterozygous mice, expressing half of the normal amount of morgana, show Rock hyperactivation in the bone marrow preceding the onset of the disease. Importantly, leukemic bone marrow cells treated ex vivo with the ROCK inhibitor fasudil, already in clinical use in Japan for the treatment of cerebral vasospasm, show a significant induction of apoptosis compared to control cells [112] (Fig. 3). The downregulation of morgana and the subsequent hyperactivation of ROCK has been found in 16% of patients with *BCR-ABL* positive CML and in 5 out of 5 patients with aCML [112]. *BCR-ABL* positive CML patients expressing low morgana levels hardly achieve the major molecular response (MMR) after 18 months of treatment with the first line tyrosine kinase inhibitor imatinib. Low morgana expression levels confer resistance to imatinib in in vitro treatment of *BCR-ABL* positive bone marrow cells from CML patients. The combination of imatinib with the ROCK inhibitor fasudil is sufficient, in vitro*,* to restore an optimal apoptotic response demonstrating that targeting BCR-ABL and ROCK signaling simultaneously could be a therapeutic strategy for BCR-ABL positive patients expressing low morgana levels. The beneficial effect of fasudil on CML and aCML patients still need to be tested.

Interestingly, JAK2, RAS and ROCK pathways are strictly interconnected: RAS binds to and activates PI3K, while JAK2 activates RAS and PI3K pathways [114] and in turn, PI3K can activate ROCK [101]. This signaling network could generate a vicious circle promoting proliferation, survival and poor treatment response in MPN. Combining inhibitors for the different network components is an interesting possibility to increase treatment efficacy and overcome resistance. Of note, a currently enrolling clinical trial (NCT02493530) will test the combination of ruxolitinib and a PI3Kδ inhibitor and aCML patients will be also enrolled in its expansion stage.

## Genetic alterations in aCML

In the past few years, scientists’ attention focused on genetic alterations characterizing aCML. The development of next generation sequencing (NGS) techniques allowed the identification of low recurrent mutations, translocations, indels and splicing variants in a number of genes [6, 70, 115–119]. These studies demonstrated that aCML is predominantly a *JAK2* V617F negative neoplasm [120, 121] with mutation in the gene found in 4% to 8% of patients [11, 14, 70]. However, other genes have been found mutated at different frequencies, mainly *ASXL1* (28%), *TET2* (16%), *NRAS* (16%), *SETBP1* (12%), *RUNX1* (12%), *ETNK1* (8%), *PTPN11* (4%), *CSF3R* (3%) [70, 115–117, 119, 122, 123]. These findings may have crucial relevance in directing personalized therapies, since aCML-associated genetic alterations could be susceptible to specific therapeutic approaches, directly targeting the mutant proteins or their associated pathways. *NRAS, JAK2* and *CSF3R* mutations, have been previously discussed and since they directly impact on the MAPK and JAK/STAT pathways, they are obvious candidates for targeted therapies. However, other two mutations are predicted to activate the signaling pathways discussed above.

*SETBP1* encodes a protein named SET binding protein 1 (SEB) for which the precise function is still to be discovered. Mutations in a particular 12 bp hot spot disrupt a degron signal, leading to SEB overexpression [124]. It has been demonstrated that SEB impacts on AKT and MAPK pathways, responsible for cell proliferation and survival [125]. In particular, SEB binds to the SET nuclear oncoprotein protecting it from protease cleavage. In turn, SET represses PP2A activity [126, 127] that inhibits AKT and MAPK pathways. When SEB is mutated, it accumulates in the cells and, through SET, decreases PP2A activity, leading to increased cellular proliferation [117].

*PTPN11* gene encodes for SHP2 (Src-homology-2 domain containing protein tyrosine phosphatase), a protein tyrosine phosphatase (PTPase) acting downstream to growth factor receptors. Mutations in the *PTPN11* gene result in constitutively activated RAS. In fact, when SHP2 is mutated it activates guanine nucleotide exchange factors (GEFs), necessary for the conversion of GDP-RAS into GTP-RAS [14, 128]. Interestingly, SHP2 is phosphorylated by JAK1 and JAK2 and the phosphorylated form of SHP2 binds to GRB2 and activates RAS [129]. Moreover, *JAK2, PTPN11* and *RAS* mutations were identified as mutually exclusive in MDS, suggesting their participation to the same pathway [130]. Given the central role of RAS mutation in MPN and the convergence of *SETBP1*, *PTPN11* and *JAK2* encoded proteins on MAPK pathway overactivation, patients carrying mutations in these genes could benefit from treatment with MEK inhibitors.

However, a number of genes mutated in aCML encodes for biosynthetic enzymes, transcription factors and epigenetic modifiers. These proteins are apparently unrelated with the signal transduction molecules previously discussed and their exact role in the onset of the pathology is still unclear.

*ETNK1*, for example, encodes an ethanolamine kinase (EKI 1) which phosphorylates ethanolamine to phosphoethanolamine in the phosphatidylethanolamine biosynthesis pathway. Two recurrent point mutations impairing the catalytic activity of the kinase have been described in *ETNK1* gene in aCML [131]. The phosphatidylethanolamine biosynthesis pathway is involved in many biochemical processes like definition of membrane architecture, anchoring of proteins to the plasma membrane, mitochondria biogenesis, autophagy and progression to cytokinesis during cell division [116, 132, 133]. Due to the fact that EKI 1 contributes to different processes in the cell, the mechanisms by which the mutant protein induces myeloproliferation have not yet been clarified.

*RUNX1* encodes the alpha subunit of the core binding factor (CBF) complex. This complex activates and represses transcription of genes involved in growth, survival and differentiation pathways in hematopoietic cells, maintaining the proper balance among different lineage progenitors [134]. This gene is recurrently mutated in a variety of hematological malignancies due to chromosomal translocations and somatic mutations. Mono- and biallelic *RUNX1* mutations have been described in aCML [14]. Some mutations cause inactivation of the protein, while others induce a dominant negative activity [135]. However, the mechanism through which the mutant *RUNX1* induces myeloid expansion is still to be understood.

The TET dioxygenases, TET1, TET2, and TET3, catalyze the transfer of an oxygen atom to the methyl group of 5-methylcytocine (5-mC), converting it to 5-hydroxymethylcytocine (5-hmC) [136, 137]. This modification, in turn, promotes locus-specific reversal of DNA methylation, impacting on DNA methylation landscape [138]. *TET2* is frequently mutated in both myeloid and lymphoid malignancies [14, 122, 139–142] resulting in a wide hypermethylation phenotype [143], but, again, the precise pathways responsible for the phenotype downstream this global genome alteration have not been dissected. The hypomethylating agent decitabine, approved by FDA for the treatment of MDS and CMML, have been tested in aCML patients (regardless of *TET2* mutational status) with some positive results, even if on small cohorts of patients, and deserves better investigations [144–147].

ASXL1 (Additional of sex combs-like 1) plays a role in the recruitment of the Polycomb Repressive Complex 2 (PRC2) to its target sequences and takes part in the complex involved in deubiquitination of histone H2A lysine 119 (H2AK119) [148, 149]. Mutations of the gene, identified in patients with AML, MPN and MDS, are associated with loss of ASXL1 expression [148]. Changes in the cell following *ASXL1* mutations include: loss of PRC2-mediated gene repression, global loss of H3K27 trimethylation (H3K27me3) and derepression of the posterior *HOXA* cluster genes, including *HOXA5–9*, known to play a role in leukemogenesis [148].

All these proteins have in common a functional pleiotropy, since they can modify the expression of hundreds of genes or the functionality of many proteins in the cell. However, it is conceivable that, among the several deregulated events and pathways, few are responsible for leukemogenesis. In this view, it would be very useful to analyze the signaling pathways known to play a role in myeloproliferation in these mutational contexts in the final attempt to exploit targeted therapies with available inhibitors. Moreover, given that two or more mutations often occur simultaneously in aCML patients [119] combination therapies with different inhibitors seems, at least in theory, a promising approach.

Recently, two studies demonstrated that the percentage of healthy people showing clonal expansion of somatic mutations associated with hematologic diseases increases with age. The authors found that clonal haematopoiesis frequently involves *DNMT3A*, *TET2*, and *ASXL1* mutant cells. Of note, somatic mutations were found to be associated with increased risk of hematological malignancies, as well as other adverse events [150, 151]. It will be tempting to envisage specific strategies for the prevention of the disease based on the mutations arising during the precancerous phases, however the predictive power of mutant hematopoiesis is low and additional biomarkers are needed to justify pharmacological intervention [150, 151].

## Conclusions

aCML is a rare hematological disease for which no standard of care exists. NGS techniques have allowed in the past few years to highlight mutations in signal transduction proteins but also in proteins with pleiotropic functions, like transcription factors and chromatin-modifying enzymes [14]. These proteins may regulate the expression of thousands of genes simultaneously, deeply altering cell physiology. However, the precise mechanisms by which they induce and sustain tumorigenesis are still elusive. In particular, it is not known whether a single gene or a specific subgroup of genes controlled by these enzymes are responsible for cell transformation and through which mechanism. It is conceivable that wide alteration in gene expression could impact on the specific signal transduction pathways regulating proliferation and survival in haematopoietic cells. However, a wide analysis of signal transduction alterations in the different mutational contexts is still missing. This information will help to identify new therapeutic approaches in genetically defined subsets of diseases, but also to successfully repurposing existing drugs. As discussed in this review, JAK2, MEK and ROCK inhibitors might represent a treatment option for aCML patients. However, apart from encouraging preclinical studies and case reports, we still need multicenter randomized trials to test the potential benefits of these treatments in large cohorts of patients.

## Abbreviations

ABL: Abelson murine leukemia viral oncogene homolog 1
aCML: Atypical Chronic Myeloid Leukemia
AKT: AKT serine/threonine kinase
ALL: Acute lymphoblastic leukemia
AML: Acute Myeloid Leukemia
ASXL1: Additional of sex combs-like 1
BCR: Breakpoint cluster region
BRAF: B-Raf proto-oncogene, serine/threonine kinase
CBF: Core Binding Factor
Chordc1: cysteine and histidine rich domain containing 1
CML: Chronic Myeloid Leukemia
CMML: Chronic Myelomonocytic Leukemia
CNL: Chronic Neutrophilic Leukemia
CSF3: colony stimulating factor 3
CSF3R: colony stimulating factor 3 receptor
DNA: deoxyribonucleic acid
EK 1: Ethanolamine Kinase 1
EPO-R: erythropoietin receptor
ERK1/2: extracellular signal-regulated kinases1/2
ET: Essential Thrombocythemia
FGFR1: fibroblast growth factor receptor 1
FLT3: fms related tyrosine kinase 3
G-CSF: Granulocyte-colony stimulating factor
GDP: guanosine diphosphate
GEFs: Guanine nucleotide exchange factors
GTP: Guanosine-5′-triphosphate
HOXA: homeobox A cluster
HRAS: Harvey RAS oncogene homolog
HSC: Hematopoietic Stem Cell
JAK2: Janus kinase 2
JMML: Juvenile Chronic Myelogenous Leukemia
JNK: c-Jun N-terminal kinase
KRAS: Kirsten RAS oncogene homolog
MAPK: mitogen-activated protein kinase
MDS: Myelodysplastic syndrome
MDS/MPN-U: Myelodysplastic/Myeloproliferative neoplasms unclassifiable
MF: Myelofibrosis
MPN: Myeloproliferative neoplasm
NGS: Next Generation Sequencing
NRAS: neuroblastoma RAS viral oncogene homolog
PDGFRA/B: Platelet Derived Growth Factor Receptor A/B
PI3K: Phosphatidylinositol-4,5-bisphosphate 3-kinase
PP2A: protein phosphatase 2, alpha isoform
PRC2: Polycomb Repressive Complex 2
PTPase: Protein Tyrosine Phosphatase
PTPN11: protein tyrosine phosphatase, non-receptor type 11
PV: Polycythaemia Vera
ROCK1/2: Rho associated coiled-coil containing protein kinase 1/2
RUNX1: runt related transcription factor 1
SEB: SET binding protein 1
SHP2: Src-homology-2 domain containing protein tyrosine phosphatase
STAT: Signal transducer and activator of transcription
SYK: spleen associated tyrosine kinase
TET1/2/3: Ten-eleven translocation methylcytosine dioxygenase 1/2/3
TPO-R: thrombopoietin receptor
WBC: White Blood Cells
WHO: World Health Organization
